# Prioritizing efficacy over convenience

**DOI:** 10.1093/ajrccm/aamag294

**Published:** 2026-06-24

**Authors:** Salman Siddiqui, Laura Brooks, Andrew Menzies-Gow, Praveen Akuthota

**Affiliations:** National Heart and Lung Institute, National Institute for Health and Care Research Imperial Biomedical Research Centre, Imperial College London, London, United Kingdom; Respiratory and Immunology, BioPharmaceuticals R&D, AstraZeneca, Cambridge, United Kingdom; Respiratory and Immunology, BioPharmaceuticals Medical, AstraZeneca, Cambridge, United Kingdom; Department of Medicine, University of California San Diego, La Jolla, CA, United States

*To the Editor*

We have read with interest the article by Dr Geoffrey Chupp and colleagues, “Switching to twice-yearly depemokimab from mepolizumab/benralizumab in severe asthma: a multicenter, randomized, double-blind, phase 3A clinical trial (NIMBLE).”^1^ This trial investigated the efficacy and safety of switching to depemokimab in participants with severe asthma already managed with and responsive to mepolizumab and benralizumab, targeting IL-5 or its receptor (IL-5/Rα), respectively.

Despite not meeting the primary endpoint of demonstrating noninferiority (predefined margin of 1.28) in annualized exacerbation rates after switching responders to mepolizumab or benralizumab to depemokimab, we noted that the manuscript concluded that “the results suggest that people with severe asthma and documented benefit from IL-5-directed biologic treatment can safely and effectively switch from a short-acting biologic such as mepolizumab or benralizumab to an ultra-long-acting biologic such as depemokimab.”^1^

A noninferiority margin is the threshold of maximum acceptable difference in effectiveness between a new treatment and a standard treatment.^2^ Therefore, the conclusion is not substantiated as noninferiority was not met in the overall population due to the upper bound of the rate ratio 95% confidence interval for clinically significant exacerbations of 1.38, exceeding 1.28, when comparing participants switched to depemokimab, with those continuing their anti-IL-5/Rα treatment: annualised exacerbation rate, 0.57 (95% CI, 0.50-0.64) vs 0.49 (95% CI, 0.43-0.55) (rate ratio = 1.16; 95% CI, 0.98-1.38) (Table 2, Chupp, et al.). Given these results, we would respectfully question the use of the wording “safely and effectively switch” in the conclusion.

Furthermore, in the prespecified subgroup analysis by prestudy biologic treatment, while there was no evidence of an increase in exacerbation rate for participants who switched from mepolizumab to depemokimab compared with those continuing on mepolizumab (rate ratio = 0.99; 95% CI, 0.78-1.26), a higher exacerbation rate was observed for participants who switched from benralizumab to depemokimab compared with those who continued on benralizumab (rate ratio = 1.38; 95% CI, 1.09-1.75; Table 3, Chupp et al.). This indicates a 38% higher rate of exacerbations in participants who switched to depemokimab, which is a larger increase than that defined as important when setting the noninferiority margin for the NIMBLE trial and considered clinically relevant. While unlikely to have been a multiplicity controlled comparison, the results suggest that nominal statistical significance could have been achieved for the benralizumab subgroup comparison as the lower confidence interval of 1.09 exceeds 1, suggesting that continued benralizumab treatment may be superior to switching to depemokimab.

The differences in results between treatment subgroups could be explained by the respective modes of action; depemokimab and mepolizumab target IL-5, whereas benralizumab targets the IL-5 receptor and has been shown to be highly effective in depleting blood eosinophils in severe asthma and eosinophilic granulomatosis with polyangiitis.^3^^,^^4^ If participants had been followed for another 52 weeks, it would have been interesting to observe whether annualized exacerbation rates would have been further affected.

We postulate that participants switched from benralizumab to depemokimab may have experienced breakthrough eosinophilic exacerbations as evidenced by results of the prospective observational MEX and BenRex studies in participants with severe refractory eosinophilic asthma. The BenRex study showed that eosinophils remained suppressed during benralizumab treatment and that exacerbations were noneosinophilic in nature.^5^ Conversely, the MEX study showed that when participants were receiving mepolizumab, approximately half of exacerbations were still eosinophilic.^6^

Overall, these data point toward a potential shared decision-making model, with the emphasis on the patient to possibly ­prioritize maximal efficacy vs every 6-month dosing for convenience.

The results of the NIMBLE trial suggest an increased risk of exacerbations when switching from benralizumab to depemokimab but not from mepolizumab to depemokimab. Further evidence is required to understand whether this may be due to ongoing eosinophilic exacerbations.

## Supplementary Material

aamag294_Supplementary_Data
